# Reasoning behind discrepancies in periodontal diagnosis and classification: A mixed‐methods analysis

**DOI:** 10.1002/jper.70083

**Published:** 2026-02-19

**Authors:** Seok‐Mo Heo, Thomas W. Oates, Se‐Lim Oh

**Affiliations:** ^1^ Department of Periodontology School of Dentistry Jeonbuk National University Jeonju South Korea; ^2^ Research Institute of Clinical Medicine Jeonbuk National University Jeonju South Korea; ^3^ Department of Advanced Oral Sciences and Therapeutics School of Dentistry University of Maryland Baltimore Maryland USA

**Keywords:** classification, diagnosis, periodontics, qualitative evaluation, quantitative evaluation

## Abstract

**Background:**

While discrepancies in periodontal diagnosis and classification among dental providers with various educational backgrounds have been reported when the 2017 Classification of Periodontal Conditions was applied, the cognitive reasoning processes underlying these discrepancies remain underexplored. This observational study investigated the underlying reasoning for errors in periodontal assessment and diagnosis by analyzing dental students’ responses to open‐ended questions (OEQs) using a mixed‐methods approach.

**Methods:**

A total of 138 dental students responded to three OEQs addressing periodontal diagnosis, risk factor identification, and periodontitis staging. Quantitative analysis using descriptive statistics assessed overall performance. Qualitative content analysis—independently conducted by two investigators—identified key periodontal diagnostic terms and reasoning trends. Alluvial plots visualized cognitive reasoning pathways.

**Results:**

Only 39 respondents (28%) correctly diagnosed a gingivitis case by integrating clinical and radiographic assessments. Fifty‐three respondents (38%) answered smoking as the appropriate risk factor for periodontal disease; the remaining included systemic diseases, etiologic and local contributing factors, and/or clinical signs along with smoking. One hundred respondents (72%) correctly assigned Stage III for a periodontitis case, which demonstrated the highest accuracy among the three tasks; the remaining showed an overestimation tendency, often skipping consideration of tooth loss and overemphasizing case complexity.

**Conclusion:**

The study findings revealed that diagnosing gingivitis was more challenging than determining periodontitis stage, with an overestimation tendency by relying on a single parameter, such as pocket depth. Difficulty in integrating clinical and radiographic data and interpreting case complexity contributed to misdiagnosis and incorrect periodontitis staging.

**Plain language summary:**

This study explored how dental students think through periodontal cases by analyzing their written responses to three OEQs on real‐world case scenarios involving periodontal diagnosis, risk factor identification, and periodontitis staging. Among 138 respondents, 28% correctly diagnosed the gingivitis case; 38% identified smoking as the risk factor; 72% correctly assigned Stage III. Common errors included overreliance on probing depth, limited use or misinterpretation of bone loss on radiographs. For staging, respondents frequently overlooked the number of teeth lost due to periodontitis and overweighted isolated periodontal complexity features, leading to overrating. These findings indicate a need for structured, case‐based training emphasizing integration of clinical and radiographic data to improve diagnostic accuracy.

## 1 INTRODUCTION

Periodontal disease is enormously prevalent. Periodontitis alone affects nearly 42% of adults aged ≥30 years in the United States.^1^ Gingivitis is also common in younger adults, although it has not been exactly measured in nationwide surveys.^2^ Combining both gingivitis and periodontitis, the overall prevalence of periodontal disease is significantly high. The 2017 World Workshop defined periodontal health and gingivitis.^3^ The workshop also outlined a comprehensive framework for the Classification of Periodontal and Peri‐Implant Diseases and Conditions by introducing the staging and grading system.^4^ This staging and grading system emphasizes key periodontal parameters such as clinical attachment loss (CAL), radiographic bone loss (RBL), the number of teeth lost because of periodontitis, case complexity, and risk modifiers.

The accuracy of periodontal diagnosis directly influences the selection of appropriate treatments, the timing of interventions, and the protocols for monitoring disease progression or stability.^5^ Misdiagnosis, whether through underestimation or overestimation of disease severity, can lead to undertreatment, overtreatment, or delayed care.^5^ Inaccurate staging may also affect the estimation of disease prognosis and communication with patients and interdisciplinary care teams.^6^ Therefore, diagnostic accuracy is not only a matter of academic excellence but also a foundation of patient care.

In the United States, most periodontal cases are managed by general practitioners.^7^ Studies have shown that general practitioners often demonstrate lower diagnostic accuracy compared with periodontists who are trained to apply the diagnostic criteria with high consistency and accuracy, when the 2017 classification system is applied.^6^, ^8^, ^9^, ^10^, ^11^ Therefore, improving diagnostic accuracy for periodontal cases among general practitioners emerges as a primary concern.

However, the aforementioned studies have largely focused on quantifying the extent of diagnostic agreements/disagreements among dental professionals with various educational backgrounds and different clinical experiences. While those studies have measured and compared diagnostic errors among their study populations, they have not explored the cognitive reasoning processes that have led to diagnostic errors. This lack of insight into critical reasoning processes presents a significant limitation to proposing further actions for improving diagnostic accuracy among general practitioners. Without understanding how general practitioners make diagnostic decisions, educational strategies intended to reduce diagnostic errors would be less effective.^12^, ^13^

To address this lack of qualitative insight into critical reasoning processes, this mixed‐methods observational study evaluated and analyzed dental students’ responses to open‐ended questions (OEQs) on periodontal diagnosis, risk factor identification, and the stage of periodontitis. The purpose of this study was to quantify the errors in periodontal diagnosis and assessment and to explore the underlying cognitive factors for these errors. The study intends to inform focused educational interventions to enhance diagnostic reasoning processes both in educational and clinical practice settings.

## 2 MATERIALS AND METHODS

This mixed‐methods study adhered to the Declaration of Helsinki and was conducted under a non‐human subject research (NHSR) protocol approved by the Institutional Review Board (IRB) at the University of Maryland, Baltimore (UMB) (HP‐00111980). Obtaining informed consent was waived by the IRB at UMB, as this study was conducted retrospectively without direct interaction with the participants. We followed the Standards for Reporting Qualitative Research (SRQR) reporting guideline.^14^

### 2.1 Participants and data collection

This study evaluated the responses from 138 second‐year dental students (Class of 2026) at the University of Maryland School of Dentistry (UMSOD) on the second‐year periodontics course final examination, which took place in March 2024. The second‐year periodontics course focused on periodontal assessment, diagnosis, classification, identification of risk factors and local contributing factors, and nonsurgical treatments for managing gingivitis and periodontitis.^15^ Course content included consideration of the 2017 periodontal classification system, with one introductory lecture and two additional review lectures, including a couple of exemplary cases, before the final examination. All of the students successfully completed the first‐year periodontics curriculum, which focuses on healthy gingiva and gingivitis, before taking the second‐year course.

### 2.2 Qualitative content analysis

A qualitative content analysis was conducted for the student responses on three constructive‐response OEQs designed to assess critical reasoning in the following areas: (1) periodontal diagnosis, (2) identification of risk factors, and (3) determination of periodontitis stage. The examination questions considered in this study were based on two distinct cases: (1) a gingivitis case for questions on periodontal diagnosis and risk factors, and (2) a periodontitis case for the determination of periodontitis staging question. The questions related to diagnosis and risk factor identification were adapted from a previously published study.^16^ For the periodontitis staging question, a table defining periodontitis staging was presented to students with the examination question, along with the comprehensive case description, which included medical, social, and dental histories, a comprehensive periodontal chart, and a complete set of intraoral radiographs. These three OEQs and their gold standard answers were internally reviewed and verified by three independent board‐certified periodontists who were not involved in this study. Full consensus was reached on question clarity and answer accuracy prior to administration.

All responses were graded by the course director (SO) using a previously published grade rubric.^16^ The course director provided the rubric before the examination, explaining that points would be assigned based on identifying the patient findings relevant to determining the diagnosis and staging, consistent with American Academy of Periodontology (AAP) guidelines. This approach was intended to evaluate recognition of critical diagnostic elements. The three questions and their corresponding expected answers are available as Supplementary Information.

The study content analysis followed the methodological framework described by Graneheim and Lundman.^17^ To enhance objectivity and minimize bias in the selection of periodontal diagnostic terms, two investigators with complementary educational and clinical backgrounds conducted the content analysis: one (SA) was a general practice educator with 2 years of Advanced General Dentistry training, and the other (SH) was a periodontist and clinical educator. Two investigators independently coded responses by extracting key periodontal terms and entering them in parallel columns in spreadsheet software* for each response. These terms were labeled with appropriate codes.

Discrepancies in the coding scheme were resolved throughout regular discussions to ensure consistency and clarity. A third investigator (SO) was consulted to resolve discrepancies if needed. The codes were clustered into categories based on their content similarity, such as pocket depth (PD), bleeding on probing (BOP), CAL, RBL, the number of teeth lost because of periodontitis, case complexities, risk factors, and other related factors.

### 2.3 Statistical analysis

All responses from the 138 participants were included in the analysis. No formal power analysis was undertaken, as the sample size was considered sufficient to support both qualitative and quantitative analyses.^18^

Descriptive statistics were used to evaluate student performance on the three OEQs, with correct and incorrect response rates calculated for each question. To identify common misconceptions and reasoning trends, the frequency of diagnostic terms used in student responses was tabulated. Alluvial plots were generated to visually represent the flow of reasoning and the distribution of diagnostic decisions across the three questions. Statistical analyses were performed using a software program.† Data visualizations were created using an open‐source tool.‡

## 3 RESULTS

Table 1 presents the correct answer rates for the three OEQs. The highest correct response rate was for the question on periodontitis stage, with 72% of students answering correctly. The lowest correct response rate was for periodontal diagnosis, with only 39 students (28.3%) diagnosing gingivitis correctly. Two students (1.4%) did not explicitly make a diagnosis, instead stating “*some probing depths are >* *3 mm and bone losses*” and “*it appears that the patient does not present with periodontal diseases*.” Ninety‐seven students (70.3%) diagnosed the case as periodontitis, using descriptors of severe, generalized, or localized. The risk factor question also had a low correct response rate of 38%, identifying smoking as the correct risk factor.

**TABLE 1:** Student performances in the three open‐ended questions (*N* = 138).

Case	Question	Correct answer	No. of students with the correct answers (%)
Case 1	Question 1. periodontal diagnosis	Gingivitis	39 (28%)
	Question 2. risk factor	Smoking	53 (38%)
Case 2	Question 3. periodontitis stage	Stage III	100 (72%)

**FIGURE 1:**
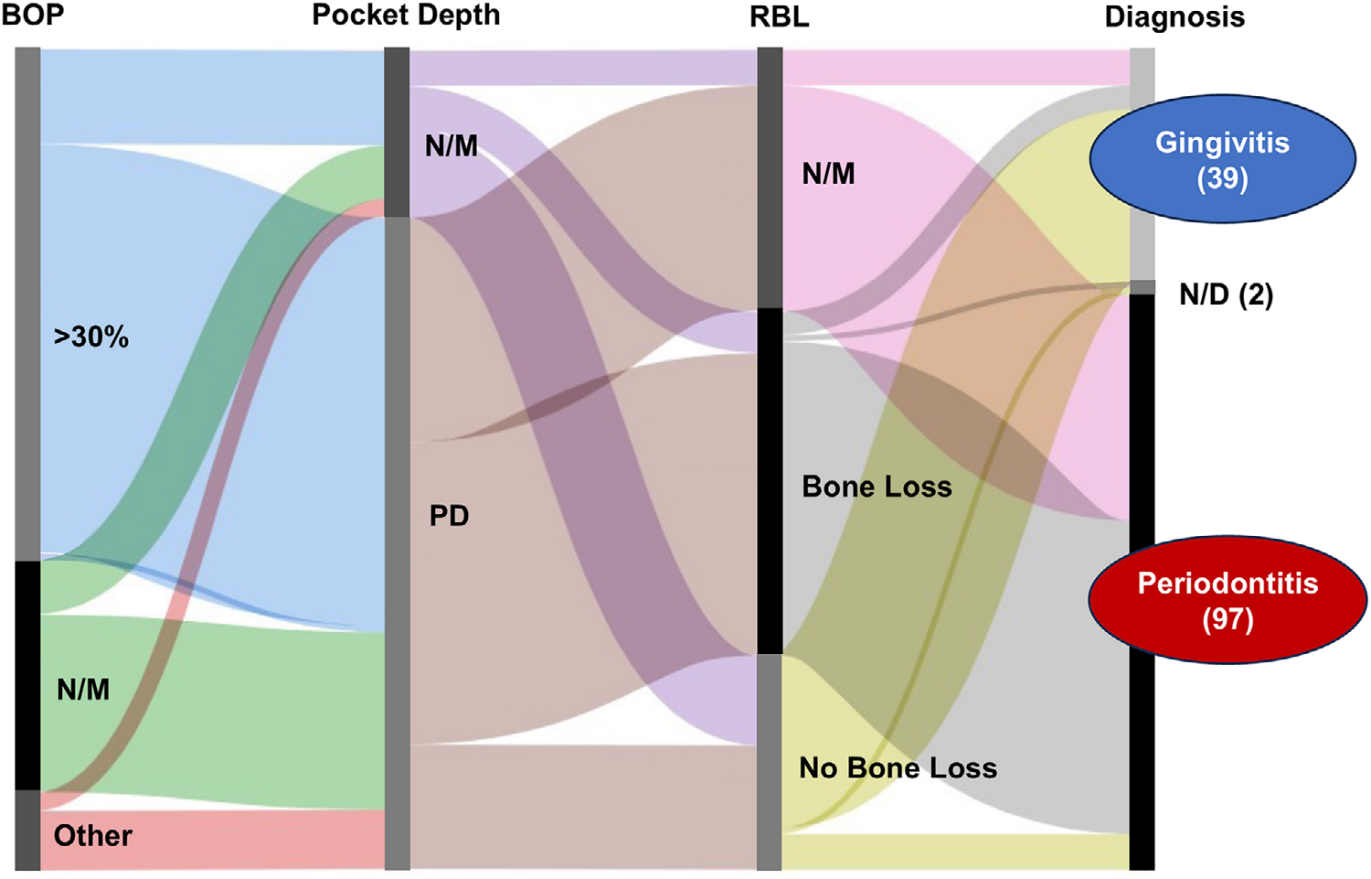
Alluvial plot for periodontal diagnosis (Case 1, Question 1, *N* = 138). BOP, bleeding on probing; N/M = not mentioned; Other included “10‐30% BOP” and “yes”; PD, pocket depth; RBL, radiographic bone loss; N/D, not determined.

In the content analysis for the periodontal diagnosis question, PD was the most frequently stated term; 80% of students mentioned PD in their responses. Other responses included BOP (72%), RBL (61%), and risk factor (45%) at lesser rates (Table 2). Figure 1 illustrates the flow of decision‐making for periodontal diagnosis. Students who focused only on PD while overlooking the absence of RBL or misinterpreting the radiographic crestal bone loss mostly misdiagnosed the case.

**TABLE 2:** Frequency of periodontal terms identified guiding periodontal diagnosis (Case 1, Question 1; *N* = 138).

Category	No. of students (%)	Correct description	No. of students with the correct description (%)	Other descriptions
PD	110 (80%)	>3 mm, high, or deep	110 (80%)	
BOP	99 (72%)	>30%	86 (62%)	10‐30% or yes
RBL	84 (61%)	No bone loss	36 (26%)	bone loss
Risk factor	62 (45%)	Smoking	30 (22%)	systemic disease and/or Crohn's disease
Plaque score	34 (25%)	Low plaque‐free score	34 (25%)	
CAL	20 (14%)	No CAL or not available	11 (8%)	CAL present

Abbreviations: BOP, bleeding on probing; CAL, clinical attachment loss; PD, pocket depth; RBL, radiographic bone loss.

**FIGURE 2:**
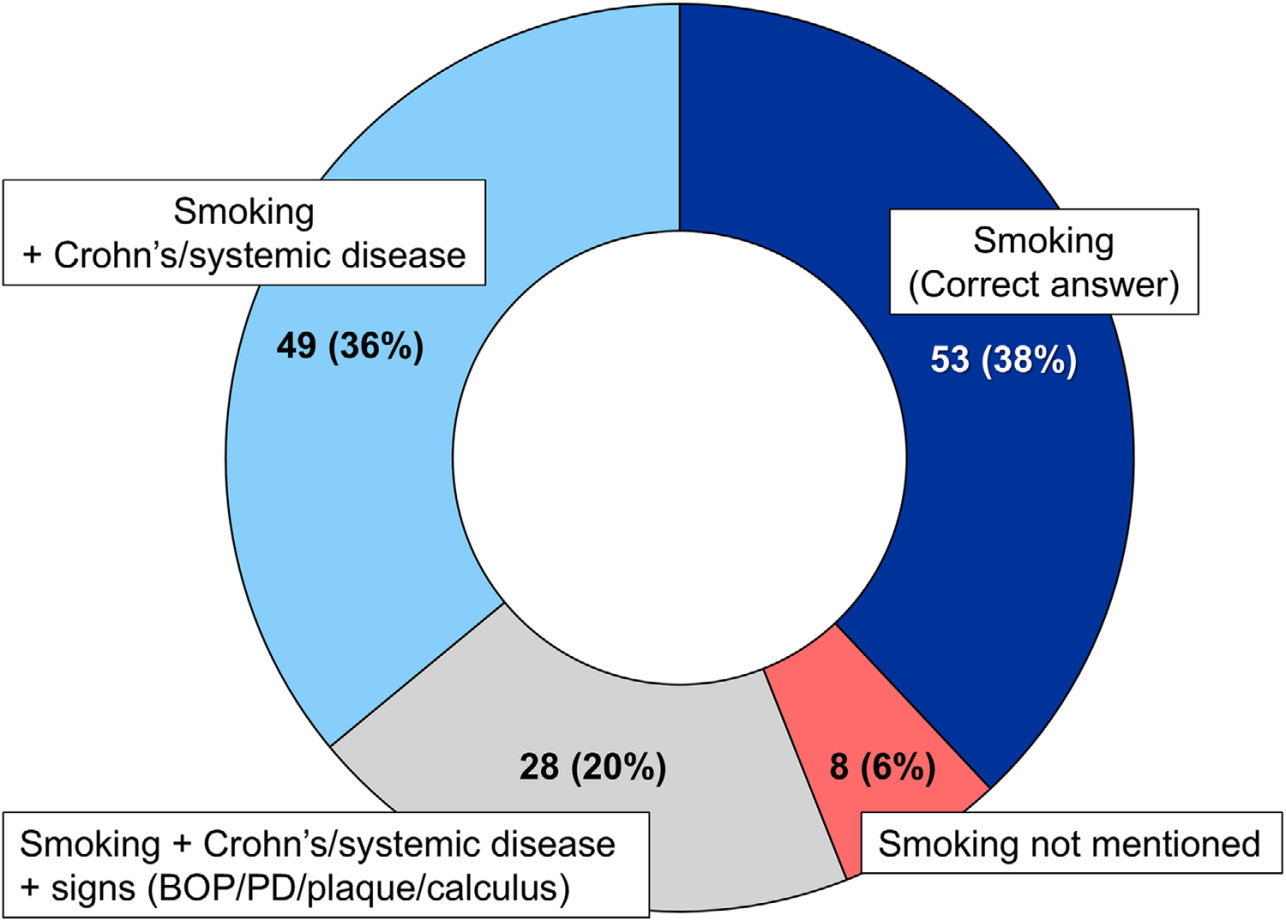
Distributions of correct and incorrect answers for identification of risk factors (Case 1, Question 2, *N* = 138). Count (%). BOP, bleeding on probing; PD, pocket depth.

Figure 2 shows the distribution of correct and incorrect answers to the risk factor question. 53 respondents (38%) answered smoking as the appropriate risk factor. 49 respondents (36%) included Crohn's disease and/or systemic disease along with smoking. 28 respondents (20%) included signs of periodontal disease (BOP and PD), etiology (plaque), and local contributing factor (calculus) in addition to smoking and/or Crohn's/systemic disease. Eight respondents (6%) did not recognize smoking as the correct risk factor for periodontal disease.

In the content analysis of responses to the periodontitis stage question, two responses were excluded because no justifications were provided. Table 3 presents the frequency of periodontal categories among the 136 responses; CAL was cited by 92% of students, followed by complexity (72%), RBL (61%), and tooth loss (51.5%). A total of 125 students referenced CAL, most accurately describing it as “ > 5 mm,” while others used terms like “ < 4 mm” or “multiple sites.” Sixteen students reasonably determined that the case had insufficient complexity for Stage IV, whereas 82 described various complexity indicators such as vertical bone loss and furcation involvement. RBL was noted by 83 students, with 77 “middle thirds” being the correct interpretation. Tooth loss was recognized by 70 students, commonly described as “ < 4” (correct), “ > 5 (incorrect),” or “missing teeth (not specified).” Sixty‐nine students noted PD.

**TABLE 3:** Frequency of periodontal categories identified guiding the periodontitis stage classification (Case 2, Question 3; *n* = 136).

Category	No. of students (%)	Correct description	No. of students with the correct description (%)	Other descriptions
CAL	125 (91%)	>5 mm	121 (89%)	<4 mm, multiple sites, middle third
Complexity	98 (72%)	Insufficient complexity for Stage IV	16 (12%)	vertical bone loss, drifting/flaring, secondary occlusal trauma, furcation involvements
RBL	83 (61%)	Middle third	77 (56.6%)	apical third, coronal third
Tooth Loss	70 (51%)	<4	67 (49%)	>5, missing teeth
PD	69 (50.7%)	Maximum PD ≥6 mm	69 (50.7%)	

**FIGURE 3:**
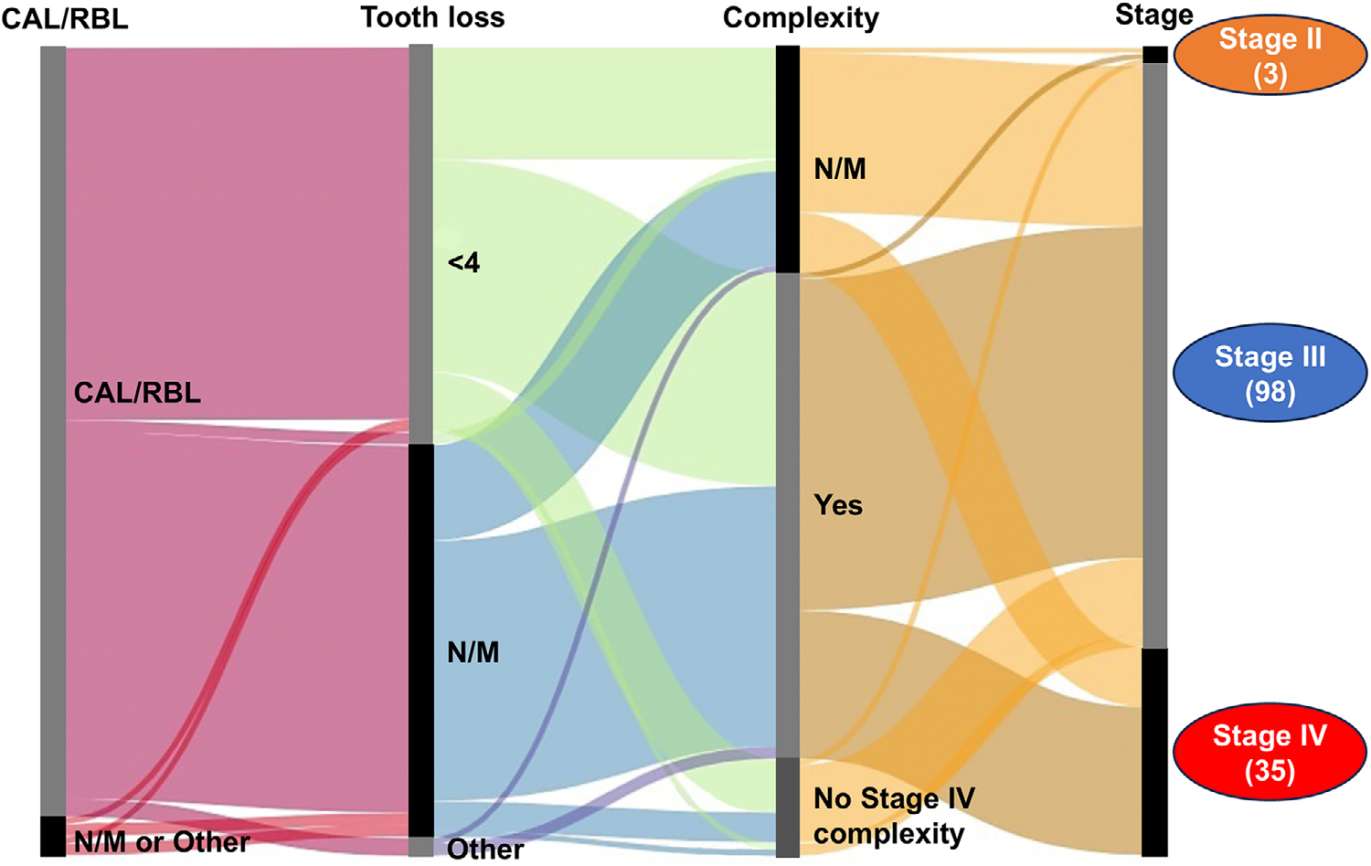
Alluvial plot for periodontitis stage (Case 2, Question 3, *n* = 136). CAL, clinical attachment loss; RBL, radiographic bone loss; N/M, not mentioned; Other included 1) CAL < 4 mm, multiple sites, or middle third, and 2) RBL coronal 1/3 or apical 1/3.

Figure 3 illustrates the decision‐making process for assigning the stage of periodontitis. Students who overlooked the amount of tooth loss and focused primarily on case complexities were more likely to assign incorrect stages, which were often overestimated (Stage IV) rather than underestimated (Stage II).

## 4 DISCUSSION

While periodontal diagnostic inaccuracies among general dentists have been consistently reported, one study found that senior dental students performed comparably to periodontists and even better than practicing general dentists in defining periodontal cases.^8^ Our study included second‐year students who were completing their preclinical education. While they exhibited a lack of clinical experience, they had received thorough didactic training. The correct response rate for periodontitis staging was 72%, close to the 76.6% accuracy among periodontal experts as reported in another study.^19^

Our respondents tended to overestimate periodontal disease, especially for a gingivitis case. As CAL was not provided in the case description, RBL should have been used as the next diagnostic key parameter to distinguish between gingivitis and periodontitis. However, most respondents stated PD as the key diagnostic parameter to support their diagnosis of periodontitis (Table 2). This content analysis indicates their lack of understanding of PD and CAL. PD alone cannot determine whether CAL exists or not. It is also important to recognize the intrinsic variability of clinical parameters, such as PD, gingival recession, CAL, and BOP, which can be influenced by examiners’ technique, patient‐specific factors, and instrument calibration.^20^ Overreliance on a single parameter without verifying evidence from objective measures, such as RBL or broader clinical context, can lead to diagnostic errors.

While combining multiple diagnostic clues is desirable,^20^ our results revealed conceptual misunderstandings about the risk factor. Forty‐five percent of respondents cited risk factors to justify their periodontal diagnosis, although the presence of risk factors alone does not confirm disease. Moreover, only 38% correctly identified the risk factor for periodontal disease. Many respondents listed systemic conditions from the case description, etiological/local contributing factors, or clinical signs as the risk factors in addition to smoking. This confusion suggests the need for clarification on the role of risk factors in periodontal assessment and diagnosis.

Respondents also struggled with applying the established criteria for periodontitis staging. Although the number of teeth lost due to periodontitis should have been considered after evaluating CAL or RBL, 49% of respondents ignored this parameter and instead focused on case complexity (Table 3). Presenting one missing mandibular first molar in this case would not demand the “complex” rehabilitation as described in the Stage IV complexity. Even if extractions were determined necessary for the two maxillary first molars with a presumable hopeless prognosis, the number of teeth lost due to periodontitis would still be <4. However, when applying complexity, many overly emphasized isolated findings, such as furcation involvement in a few teeth or a single mesially tilted tooth, rather than assessing the overall complexity of the dentition, as would be consistent with Stage IV. Additionally, some respondents may have interpreted “periodontal findings” narrowly, leading to omission of relevant severity or complexity factors essential for distinguishing between Stages III and IV.

These findings suggest that the criterion of tooth loss “because of periodontitis” remains confusing. While identifying missing teeth is straightforward, determining the cause of tooth extraction is challenging. Clarifications regarding the use of the new classification recommend including hopeless teeth based on severe periodontal destruction in counting tooth loss due to periodontitis.^21^ However, obtaining accurate dental history is often difficult, as most patients may not remember the reasons for previous tooth extractions. Moreover, factors such as the extent of caries and the severity of endodontic infections significantly affect the prognosis of individual teeth, besides or in addition to periodontal status.^22^, ^23^, ^24^

While the impact of overestimation/underestimation of periodontitis stage has not been systematically studied, such misclassification may not remarkably alter overall treatment plans. All periodontitis cases, regardless of their stages, require scaling and root planing as a part of initial therapy. In cases of teeth with a hopeless prognosis, extractions—rather than periodontal therapy—may be appropriate.

Meanwhile, overestimation/underestimation of periodontal diseases can lead to inappropriate clinical decisions, as treatment approaches for gingivitis and periodontitis differ. Misdiagnosing gingivitis as periodontitis may result in overtreatment, while failing to diagnose periodontitis could allow disease progression by delaying necessary treatments. Therefore, greater emphasis should be placed on accurate diagnosis of periodontal diseases for timely, effective treatment planning.

While the absence of clinical exposure in this study population may have contributed to difficulty in applying diagnostic frameworks to realistic case scenarios, the role of clinical experience in improving diagnostic accuracy remains arguable. Some studies report that accumulated experience enhances pattern recognition and decision‐making efficiency so that clinicians can identify familiar presentations more rapidly and intuitively,^25^ while another study indicates that clinical experience alone does not always lead to improved diagnostic performance.^26^ Diagnostic accuracy appears to be more closely linked to the quality of data collection and interpretation.^27^

There are several limitations to applying the study findings to other institutions and general practitioners. First, the study was conducted with one class that was completing preclinical education from one academic institute. In our study, students received instruction primarily through lectures that included case examples, without real patient encounters. As such, the results may not capture variability in curricular approaches in other institutes. Second, the diagnostic decisions were made during the examination, where respondents were motivated by grades and had sufficient time to think. The academic setting is different from real‐world clinical settings, where time limits, demands from patients, or unknown distractors may significantly influence clinicians’ reasoning and decision‐making processes. In addition, practicing general dentists rely more on experiential knowledge. Third, as a cross‐sectional approach, our study could not verify whether fourth‐year students or practitioners would demonstrate higher diagnostic accuracy. Therefore, the generalizability to more advanced learners is limited. Future mixed‐methods studies should apply a longitudinal assessment approach, enroll practicing general dentists, and be conducted in routine practice settings to establish educational strategies, which can be adapted to support both students and practicing general dentists.

## 5 CONCLUSION

The mixed‐methods study results revealed that diagnosing gingivitis was more challenging than determining the periodontitis stage. A lack of skills in integrating clinical and radiographic data may contribute to incorrect diagnosis. Overemphasis on case complexity, along with skipping consideration of tooth loss, led to inaccuracy in assigning the periodontitis stage.

## AUTHOR CONTRIBUTIONS

*Methodology*: Seok‐Mo Heo, Se‐Lim Oh. *Writing—review and editing*: Seok‐Mo Heo, Thomas W. Oates, and Se‐Lim Oh. *Investigation*: Seok‐Mo Heo. *Visualization*: Seok‐Mo Heo and Se‐Lim Oh. *Conceptualization*: Thomas W. Oates and Se‐Lim Oh. *Formal analysis, writing—original draft, supervision, and project administration*: Se‐Lim Oh

## CONFLICT OF INTEREST STATEMENT

The authors declare no competing financial interests or personal relationships that could have appeared to influence the work reported in this paper.

## Supporting information



Supporting information

## Footnotes

* Microsoft Excel, Microsoft Corporation

† Jamovi (Version 2.5.5) Sydney, Australia

‡ RAWGraphs, https://www.rawgraphs.io/
