# Supplementary material for: Reasoning behind discrepancies in periodontal diagnosis and classification: A mixed‐methods analysis
Source: J Periodontol. 2026 Feb 19;97(6):1351–9. doi: 10.1002/jper.70083 (PMC13350512; doi:10.1002/jper.70083)
Supplement: Supplementary file 1 — Supporting information [file JPER-97-1351-s001.pdf]

1. Based on clinical and radiographic evaluation, **make a periodontal diagnosis** for this patient. List your periodontal findings that support your decision. (3 points; 30 words)

Generalized gingivitis due to (1) 68% BOP and (2) no crestal bone loss evident on radiographs

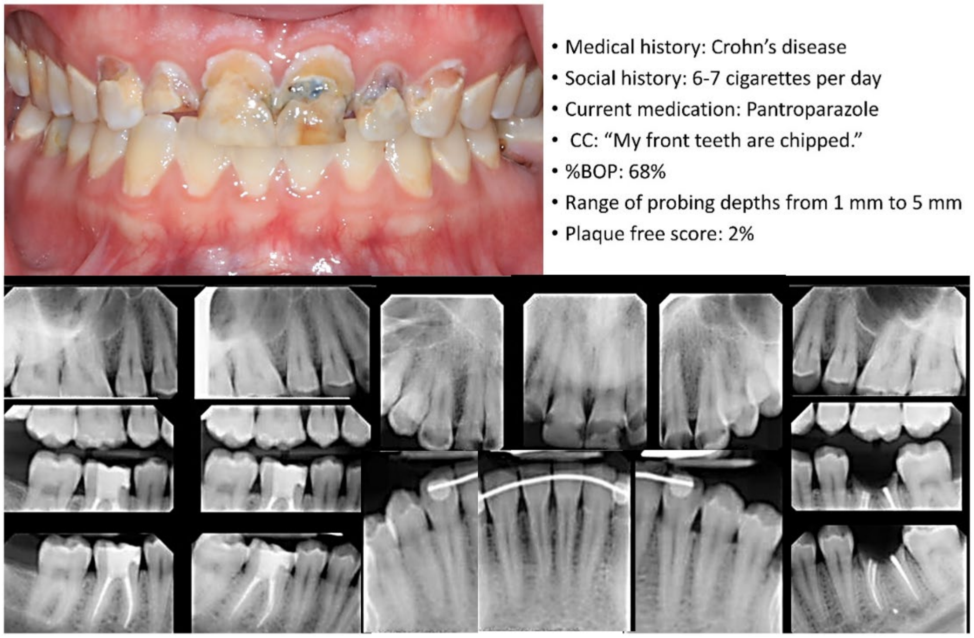

2. Does this patient have any risk factors for periodontal disease? If yes, indicate the risk factor(s). (2 points: 10 words)

Yes, he is a smoker.

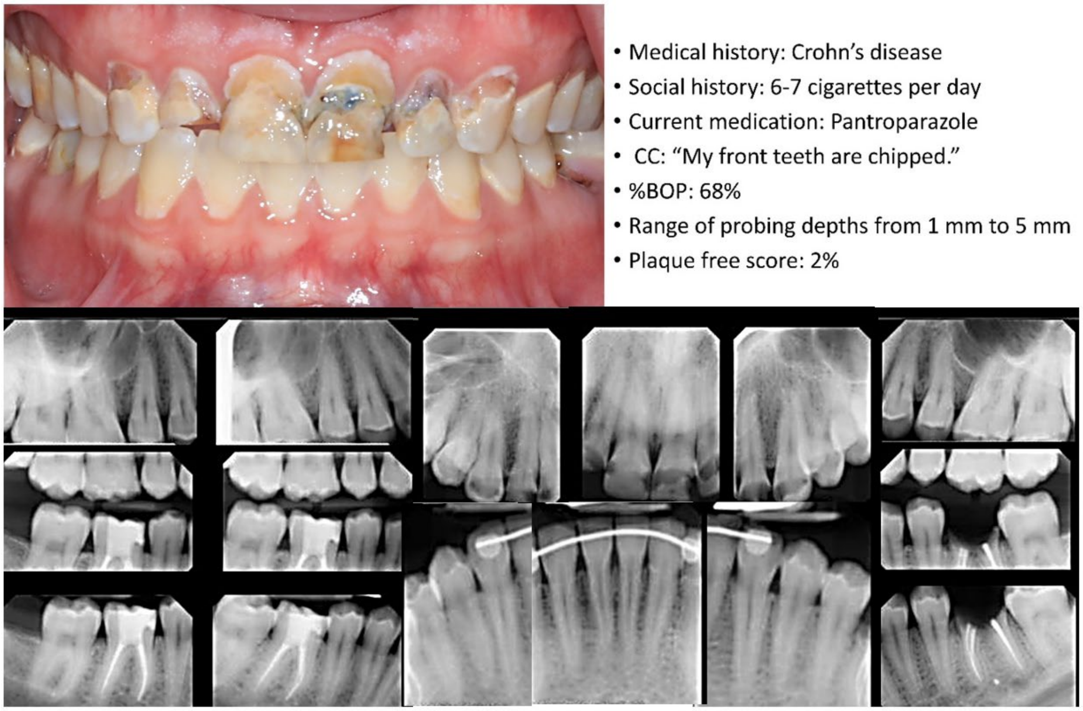

3. Based on clinical and radiographic evaluation, **assign a stage** using the 2018 periodontitis classification for this patient. List your periodontal findings that support your decision on the stage. (3 points; 30 words)

Stage III is based on more than 5 mm CAL and only one tooth loss.

- 54 y/o African American Female
- CC: "I want to clean my teeth"
- Medical Hx: Controlled Hypertension – takes nifedipine and chlorthalidone
- Social Hx: Never smoked
- Family Hx: Father had Type II diabetes
- Reports brushing twice a day for two minutes
- Flossing occasionally
- Uses mouth wash daily

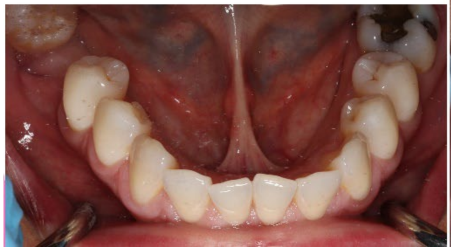

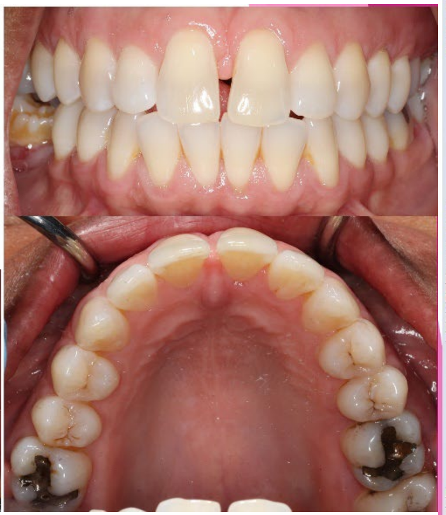

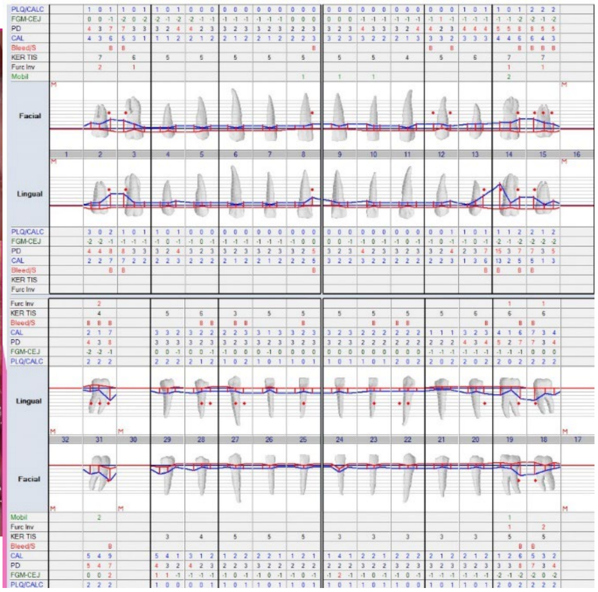

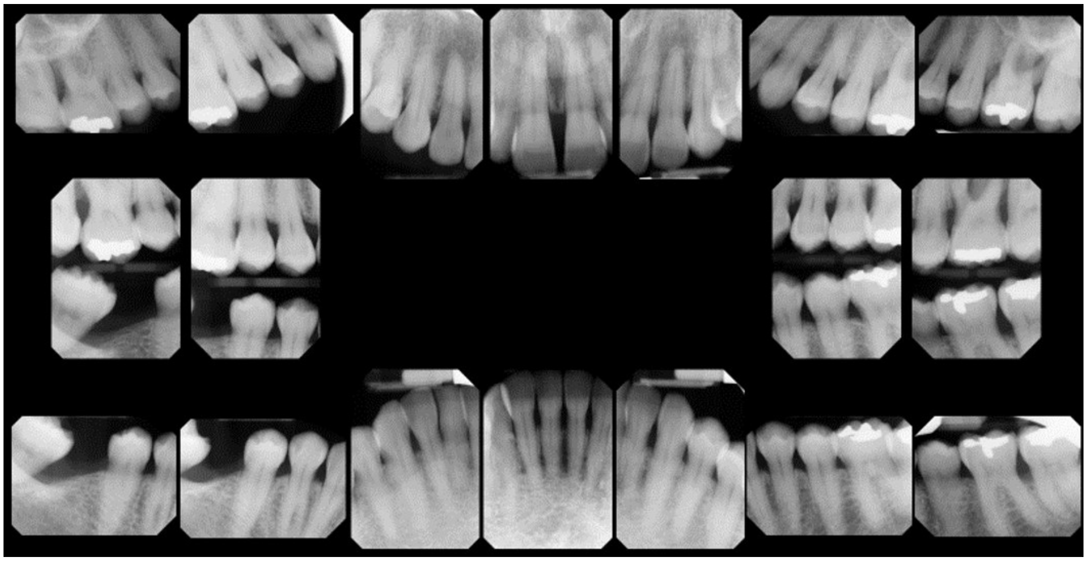

Staging	Periodontitis	Stage 1	Stage 2	Stage 3	Stage 4
Severity	Interdental **CAL (at the site of greatest loss)**	1-2 mm	3-4 mm	≥5 mm	≥5 mm
	**RBL**	Coronal third (<15%)	Coronal third (15%-33%)	Extending to the middle third of the root and beyond	Extending to the middle third of the root and beyond
	Tooth loss (due to periodontitis)	No tooth loss	No tooth loss	≤4 teeth	≥5 teeth
Complexity	Local	• Maximum PD ≤4 mm<br>• Mostly horizontal BL	• Maximum PD ≤5 mm<br>• Mostly horizontal BL	In addition to Stage 2 complexity<br>• Maximum PD ≥6 mm<br>• Vertical BL ≥3 mm<br>• FI Class II or III<br>• Moderate ridge defects	In addition to Stage 3 complexity<br>• Need for complex rehabilitation due to:<br>- Masticatory dysfunction<br>- Secondary occlusal trauma<br>- Severe ridge defects<br>- Bite collapse, drifting, flaring<br>- <20 remaining teeth (10 opposing pairs)
